# Development of a Mobility Diet Score (MDS) and Associations With Bone Mineral Density and Muscle Function in Older Adults

**DOI:** 10.3389/fnut.2019.00114

**Published:** 2019-09-04

**Authors:** Simon Rønnow Schacht, Mads Vendelbo Lind, Kenneth Hudlebusch Mertz, Jacob Bülow, Rasmus Bechshøft, Grith Højfeldt, Aide Schucany, Morten Hjulmand, Chiara Sidoli, Søren Binder Andersen, Mikkel Jensen, Søren Reitelseder, Lars Holm, Inge Tetens

**Affiliations:** ^1^Department of Nutrition, Exercise and Sports, Vitality - Centre for Good Older Lives, University of Copenhagen, Copenhagen, Denmark; ^2^Department of Orthopaedic Surgery M, Institute of Sports Medicine, Bispebjerg Hospital, Copenhagen, Denmark; ^3^School of Medicine and Surgery, University of Milano-Bicocca, Milan, Italy; ^4^Department of Biomedical Sciences, Faculty of Health and Medical Sciences, University of Copenhagen, Copenhagen, Denmark; ^5^School of Sport, Exercise and Rehabilitation Sciences, University of Birmingham, Birmingham, United Kingdom

**Keywords:** elderly, nutrition, diet, dietary pattern, bone, BMD, muscle, strength

## Abstract

**Introduction:** Reduced bone mineral density (BMD) and muscle function is associated with increased risk of multiple health related issues. Diet may play a role in sustaining BMD and muscle function throughout old age, but much is still to be learned with regards to which specific food groups and dietary patterns that are important for such outcomes. The aim of the current study was to identify food groups important for both BMD and muscle function.

**Methods:** A narrative review was performed on studies published on dietary patterns and their association with BMD and muscle function, respectively. Based on these findings, two dietary indices were constructed characterizing food groups associated with BMD and muscle function, respectively. Associations between adherence to these indices and BMD and muscle function were then investigated in a population of older community-dwelling Danes. Food groups found to be associated with both BMD and muscle function in our study population were suggested for inclusion into a common dietary index named the Mobility Diet Score.

**Results:** In contrast to previous studies, adherence to a dietary index based on foods previously linked to BMD could not be established as important for BMD in our study population of 184 older individuals (53.3% men). We found that adhering to a dietary index characterized by higher intakes of whole grains, dairy products, fish, legumes, nuts, fruit, and vegetables is associated with faster 400 m walking speeds and an increased number of chair stands measured over a 30 s time period. Since no food group could be established as important for both BMD and muscle function in our study population, a Mobility Diet Score could not be established. However, based on our narrative review, the food groups commonly associated with improved BMD and muscle function are similar.

**Conclusion:** Adherence to a dietary index characterized by high intakes of whole grains, dairy products, fish, legumes, nuts, fruit, and vegetables was not found to be associated with BMD in a group of community-dwelling older Danes. However, our results indicate that the adherence to such foods could be important in sustaining physical function in older individuals.

## Introduction

Loss of bone mineral density (BMD) as well as age-related loss of muscle mass and strength (sarcopenia) are potential concerns of older individuals. Both osteoporosis and sarcopenia are associated with adverse health outcomes such as increased risk of falls, bone fractures, hospitalization, disability, poor quality of life and death (1–5). As both bone and muscle deterioration are often correlated in older individuals, the broader term “Osteosarcopenia” has been suggested (6). Furthermore, osteoporosis and sarcopenia are potentially interrelated as osteoporosis is likely to affect physical activity level and thereby muscle maintenance and vice versa. Practicing regular physical activity helps to preserve BMD as well as skeletal muscle mass and strength in older adults (7–9). Moreover, it has been widely demonstrated that diet can positively influence the maintenance of BMD as well as functional capabilities (10–12). However, the specific dietary patterns and food groups that may be important for such outcomes are not yet well-established.

Previously, a diet score that characterizes food groups associated with BMD was developed by de Jonge et al. (13). Based on a narrative review of 15 observational studies investigating associations between different dietary patterns and BMD, eight food groups were identified and included in a BMD-Diet Score. Adherence to this diet score was subsequently found to be associated with BMD in a cohort of +5,000 Dutch older adults (median age 67 years). Other studies have found associations between dietary patterns that are abundant in whole grains, fruits, and vegetables, e.g., dietary patterns inspired by the “Mediterranean” and “healthy Nordic” diets and skeletal muscle mass and strength (14, 15). A recent systematic review of observational studies concluded that the evidence for an association between a range of physical outcomes and adherence to dietary patterns characterized by high intakes of fruit, vegetables, whole grains, fish, lean meat, low-fat dairy, nuts, olive oil and low in refined grains, sweets, and animal products was considered strong and consistent for physical performance, but limited for muscle strength and sarcopenia in older individuals (11). As low BMD can potentially lead to bone fractures, it is an important factor to consider along with muscle strength and functional capabilities as a way to maintain the quality of life and independence of older people. Establishing a dietary index that considers all of these factors is therefore highly relevant. The development of such an index seems increasingly important as the prevalence of both osteoporosis and sarcopenia likely is going to increase significantly in the years to come due to a growing global older population (16).

The aim of the present study was to investigate the food groups most commonly associated with BMD, muscle strength and physical function. Subsequently, we aimed to confirm these associations in a population of older Danish community-dwelling individuals.

## Methods

In order to establish the food groups important for BMD and muscle outcomes, the current study took a two-step methodological approach. Step 1 consisted of a narrative review of the existing literature. Here we identified the food groups that are most strongly associated with BMD and muscle mass, strength and function, respectively (all muscle related outcomes are collectively referred to as “muscle function” in this study). In step 2 we subsequently assessed the potential associations between adherence to the identified food groups and BMD and muscle function, respectively, in a group of older Danish individuals. Food groups confirmed as important for both BMD and muscle function would then be suggested for implementation into a cumulative Mobility Diet Score (MDS) representing a dietary pattern beneficial for both bone and muscle “health”.

### Study Setup

To investigate associations between the intake of food groups identified in the narrative review and BMD and muscle outcomes, the present study used baseline data from the “Counteracting Age-related Loss of Skeletal Muscle Mass” (CALM) study. The CALM study is a 1 year randomized controlled trial investigating effects of diet and physical activity on functional outcomes such as BMD, muscle size, -strength, and -performance in Danish community-dwelling older adults. The CALM study has been described in detail elsewhere (17).

#### Study Population

One hundred and eighty four Danish men and women (≥65 years) participating in the CALM study (17) were included in the present study. Only participants with complete baseline dietary registrations were included (184 out of 205).

#### Assessment of Dietary Intake

The enrolled participants completed 3 days weighed food diaries from Wednesday to Friday. Dietary information was entered into the online dietary registration tool VITAKOST™ (MADLOG ApS, Kolding, Denmark) where average daily consumption of the different food products was calculated. Consumed foods were divided into the respective food groups identified as relevant in our narrative review for use in later analyses investigating associations with BMD and muscle function.

#### Assessment of Outcomes

##### Bone Mineral Density (BMD)

Whole body, femur neck, total femur, and L1-L4 BMD measurements were obtained by dual energy x-ray absorptiometry (DXA) using the enCORE v.16 software [Lunar iDXA; GE Medical Systems, Pewaukee, WI (USA)]. BMD values are presented as g/cm^3^.

##### Muscle Function and Lean Body Mass

*30-s chair stands (30 s-cs)* A 30 s-cs was used as a measurement of physical function. Participants were instructed to stand up from a seated position with their hands crossed on the chest as many times as possible within a 30 s period. The final number of stands was then registered.

*400 m gait time (400 m-gt)*. A 400 m-gt test was performed by study participants as a measure of walking endurance. Participants were instructed to walk 400 m as fast as possible on a 20-m course marked with two colored cones without personal support or sitting down. Up to 1 min of standing was permitted if the participant felt tired or experienced discomfort, as long as the test was completed within 15 min. Time of completion was then registered.

*Hand grip strength (HGS)*. HGS was used as an indicator of strength and of potential functional limitations. HGS was examined by a handgrip strength dynamometer (DHD-1[SH100]; SAEHAN Corporation, Changwon City, South Korea) using study participant's dominant hand. A minimum of three attempts was performed with at least 30 s rest in between sets. The highest value at the given time point was used. The test was finished when one measurement was lower than the peak value.

*Knee extensor MVC*. Study participant's maximal isometric thigh strength was measured as an indicator of lower extremity strength. Maximal voluntary contractions (MVC) were performed at a knee angle of 70° flexion (0° = full knee extension) using an isokinetic dynamometer (Kinetic Communicator, model 500-11, Chattanooga, TN, USA). Participants performed three attempts, and the highest attained peak torque was registered.

*Lean body mass was measured by DXA*. Using the enCORE v.16 software (Lunar iDXA; GE Medical Systems, Pewaukee, WI (USA).

##### Assessment of Covariates

Personal information such as age and sex were registered at baseline visit to the study center. Body weight in light clothing as well as height without shoes were measured by trained healthcare personal. Physical activity was measured over a 4 day period by an accelerometer-based activity monitor (activPal 3™, activPal 3c™, or activPal micro™; PAL technologies, Glasgow, UK). Calcium, alcohol and total energy intake were calculated based on previously described dietary registrations.

### Narrative Literature Review

Two researchers conducted an independent narrative literature review on PubMed searching for articles related to dietary patterns, BMD, osteoporosis and muscle strength and functional outcomes. The search for additional studies related to BMD was performed on articles published between March 2015 [end of search, (13)] and December 2018. The search for studies including muscle outcomes was performed on all articles published in PubMed until December 2018. Search string used in PubMed: (“diet” OR “dietary pattern” OR “diet score” OR “food group” AND “bone” OR “BMD” OR “osteoporosis” OR “muscle” OR, “lean mass,” “strength” OR “musculoskeletal” OR “sarcopenia”).

The review included studies investigating pre-defined dietary patterns as well as studies investigating exploratively derived dietary patterns. Studies investigating exploratively derived dietary patterns were included only when the contribution of the specific foods was reported as factor loadings. Foods with a factor loading above or below +0.3 and −0.3, respectively, were included in the index. To account for food synergy and complex diet-disease relations (18), studies investigating single nutrients were not included in this search.

#### Development of the Mobility Diet Score (MDS)

To establish food groups relevant for preservation of both BMD and muscle function, a dietary index including food groups associated with BMD and a dietary index including food groups associated with muscle function were constructed, respectively. Similarities between these two indices were compared and food groups evaluated as relevant for both BMD and muscle function were suggested for inclusion into a common dietary index named the Mobility Diet Score (MDS).

##### Food Groups Related to BMD (Updating the BMD-Diet Score)

To establish food groups relevant for BMD the “BMD-Diet Score” constructed by de Jonge et al. (13) was updated in the present study. Similar to the original BMD-Diet Score, our updated version (BMD-DS) includes food groups commonly associated with BMD in either positive and negative directions. In the current review update, food groups investigated in relevant studies, but not included by de Jong et al. or published after March 2015 were added to the narrative review results of de Jong et al. (13). In order to establish relevant food groups, the cumulative number of times a food group was associated with BMD in the identified studies was registered. Food groups >25th percentile of this cumulative count were considered as relevant and included in the BMD-DS. A food group was included as a positive contributor to the BMD-DS if >50% of the identified studies reported a positive association with BMD outcomes, and vice versa for negatively associated food groups. Dietary intake expressed in food groups was divided into quartiles and CALM study participants were scored according to adherence. Positively associated food groups contributed positively to the BMD-DS (scoring ranging from 0 to 3 for each food group) and negatively associated food groups contributed negatively to the BMD-DS (scoring ranging from 0 to −3 for each food group).

##### Food Groups Related to Muscle Function

To identify food groups relevant for muscle function, a similar review approach as the one described above for BMD was performed. A literature search for food groups associated with muscle function and the construction of a Muscle and Functional Diet Score (MF-DS) including food groups relevant for muscle function was conducted. Inclusion of relevant food groups was established by investigating the frequency of which a food group was associated either positively or negatively with muscle function in published studies. Food groups were included into the MF-DS with either a positive or negative contribution if >50% of the identified studies reported an association between the respective food group and any relevant muscle outcome (muscle mass, strength, or function). Scoring was conducted in a similar way as for the BMD-DS with scores ranging from 0 to 3 and 0 to −3 for positively and negatively associated food groups, respectively. Similarly to the de Jonge review, alcohol intake was not included in either the BMD-DS or the MF-DS, but was instead considered a potential confounder (19, 20).

Food groups evaluated to be important in regards to both BMD and muscle function were suggested for inclusion into the MDS.

#### Statistical Analyses

Descriptive statistics are presented as medians and respective inter quartile ranges (IQR). Tests for associations between index scoring and outcomes related to BMD and muscle function were performed in the CALM study population via multiple linear regression adjusted for available confounders. Model 1 (adjusted for age, sex, and total energy intake), model 2 (adjusted for model 1 + body weight and height), and model 3 (adjusted for model 2 + physical activity level and alcohol intake). As calcium intake potentially explains (some) of the association between diet and BMD, analyses including BMD outcomes were performed with and without adjustment for calcium. Regression coefficients for the investigated associations are presented per SD as well as per quartile of intake (with Q1 as the reference) along with 95% confidence intervals (95% CI). Statistical significance was considered as *p* < 0.05. All analyses were performed in R, version 3.5.1 (21). To identify potential interactions between investigated variables, sex, age and physical activity were included as product terms in the model.

## Results

### Characteristics and Dietary Intake in the CALM Study Population

General characteristics are presented in Table 1. Briefly, the median age of the study population was 70 years, median weight was ≈ 79 and 67 kg for men and women, with a median BMI of 25 and 24 kg/m^2^, respectively. Median HGS was ≈ 46 and 27 kg and 400 m-gt ≈ 230 and 245 s, respectively. Median whole body BMD was ≈ 1.28 and 1.0 g/cm^2^ for the two sexes and femur total BMD was ≈ 1.1 and 0.9 g/cm^2^, respectively.

**Table 1:** Characteristics of the CALM study population.

**Characteristics**	**Median (IQR)**		
	**Men (*n* = 98)**	**Women (*n* = 86)**	**All (*n* = 184)**
Age (year)	69.0 (6.0)	70.0 (5.8)	69.0 (6.0)
Weight (kg)	79.1 (13.7)	66.7 (15.0)	73.7 (16.1)
BMI (kg/m^2^)	25.0 (4.2)	24.0 (5.7)	24.7 (5.0)
Waist circumference (cm)	94.0 (13.8)	85.0 (20.0)	92.0 (16.5)
Blood pressure, systolic (mmHg)	144.0 (25.8)	141.5 (21.8)	142.5 (24.5)
Blood pressure, diastolic (mmHg)	85.0 (15.5)	82.0 (11.8)	83.0 (13.3)
400 m walk time (s)	230.0 (37.5)	245.5 (42.0)	240.0 (40.5)
30 s chair stands (stands)	21.0 (7.0)	18 (6.8)	20 (7.0)
Grip strength (kg)	45.9 (16.2)	27.4 (21.1)	36.5 (19.3)
Knee extensor MVC (Nm)	200.9 (64.6)	134.2 (76.3)	163.9 (69.7)
Whole body BMD (g/cm^2^)	1.28 (0.24)	1.0 (0.29)	1.17 (0.27)
Femur neck BMD (g/cm^2^)	0.95 (0.18)	0.83 (0.19)	0.90 (0.20)
Femur total BMD (g/cm^2^)	1.10 (0.24)	0.88 (0.24)	0.96 (0.23)
L1–L4 BMD (g/cm^2^)	1.31 (0.26)	1.13 (0.25)	1.24 (0.32)

*IQR, inter quartile range (Q3–Q1); BMD, bone mineral density; MVC, maximum voluntary contraction*.

The median food intake (g/d/10MJ) in the study population is presented in Table 2. Large variations were observed for the intake of most foods, exemplified by meat and alcohol intake ranging from total abstainers (intake = 0 g) to intakes >500 and 80 g per day, for the two food groups, respectively. The food intake in this study population is resembling that of the latest national Danish dietary survey of older men and women (22), but with higher intakes of vegetables and coarse grain products.

**Table 2:** Median dietary intake in the CALM study population, g/d/10MJ.

**Food group**	**Median (IQR)**		
	**Men (*n* = 98)**	**Women (*n* = 86)**	**All (*n* = 184)**
Coarse grains	138.5 (120.3)	136.6 (100.7)	138.5 (102.9)
Refined grains	104.1 (106.7)	60.3 (99.6)	81.3 (116.9)
Meat and meat products	116.2 (130.4)	96.0 (90.7)	104.2 (116.6)
Poultry and poultry products	22.5 (41.3)	21.0 (31.0)	21.8 (36.2)
Fish and seafood	55.9 (81.5)	55.8 (71.6)	55.8 (79.2)
Egg	19.7 (45.6)	26.5 (48.5)	21.4 (48.0)
Fruits	120.6 (208.8)	253.9 (281.9)	182.2 (233.5)
Vegetables	514.2 (479.0)	620.5 (383.8)	574.9 (454.6)
Dairy products	251.6 (301.9)	351.7 (253.0)	308.1 (305.1)
Legumes and nuts	29.2 (22.2)	23.7 (25.7)	26.6 (23.2)
Vegetable oils	4.6 (12.6)	11.3 (14.7)	7.3 (17.0)
Butter and other fats	12.8 (34.4)	8.2 (22.0)	10.4 (30.5)
Confectionery	51.6 (88.3)	60.9 (79.0)	57.0 (84.0)
Coffee and tea	563.9 (465.6)	708.6 (628.6)	618.8 (555.7)
Alcohol	16.0 (22.4)	11.1 (19.0)	13.5 (20.7)

*IQR, inter quartile range (Q3–Q1)*.

### Narrative Review and the Identification of Food Groups Associated With BMD and Muscle Function in Previously Published Studies

In total, seven additional papers were identified investigating associations between dietary patterns and BMD (23–29). All seven studies included dietary patterns identified via exploratively-derived methods in the form of either PCA, factor analyses or similar. A short description of the included studies and the investigated dietary patterns are presented in Tables S1, S2.

Our search revealed nine studies investigating associations between dietary patterns and outcomes related to muscle function. Three of these studies included dietary patterns exploratively derived via PCA or factor analysis (30–32). The remaining six studies included pre-defined dietary patterns (14, 15, 33–36). A short description of the included studies and the investigated dietary patterns are presented in Table S3.

**Figure 1:**
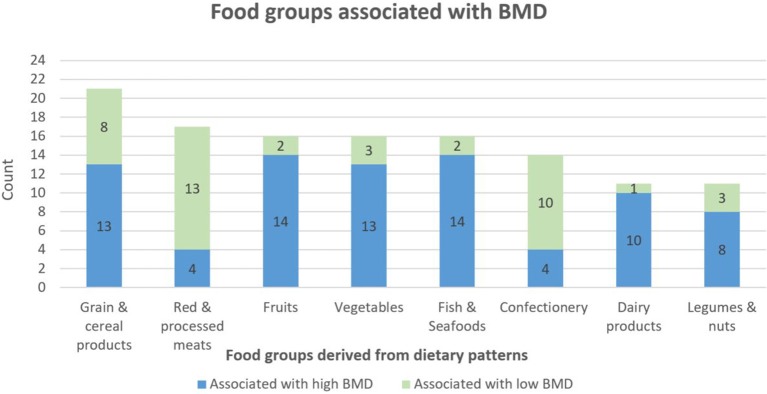
Food groups associated with bone mineral density (BMD). Count equals the number of times a food group was associated with BMD in our narrative review (only food groups with a cumulative count >25th percentile is shown). Update of de Jonge (13) review. Green color indicates that a food group was found to be associated with low BMD in one or several studies in the literature. Blue color indicates that a food group was found to be associated with high BMD.

Based on our narrative review, eight food groups were identified as relevant for BMD and included in our updated version of the BMD-Diet score. These were “grain and cereal products,” “red and processed meats,” “fruits,” “vegetables,” “fish and seafood,” “confectionery,” “dairy products,” and “legumes and nuts” (Figure 1). Based on our study criteria, red and processed meats and confectionery were the only food groups found to be negatively associated with BMD and hence, the only food groups included in our BMD-DS as negative contributors.

**Figure 2:**
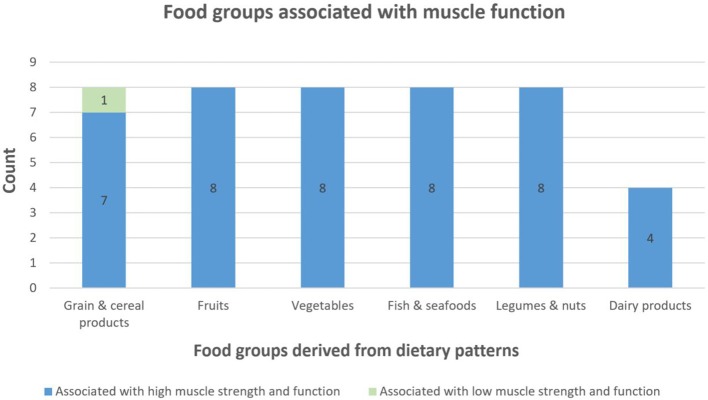
Food groups associated with muscle function. Count equals the number of times a food group was associated with outcomes related to muscle function in our narrative review (only food groups with a cumulative count >25th percentile is shown). Green color indicates that a food group was found to be associated with low BMD in one or several studies in the literature. Blue color indicates that a food group was found to be associated with high BMD.

Six food groups were identified as relevant for muscle function and therefore included in the MF-DS. These were “grain and cereal products,” “fruits,” “vegetables,” “fish and seafood,” “dairy products,” and “legumes and nuts” (Figure 2).

The grain and cereal food group were in the present study divided into coarse and refined grain products as primarily whole grain foods were found to be positively associated with the investigated BMD and muscle function and vice versa for refined grain products. Most of the identified studies investigated Mediterranean and Healthy Nordic diets in relation to muscle function (14, 33, 35, 36). Although meat intake is scored negatively in these dietary patterns and should hence contribute negatively to our index, we did not include meat as a negative contributor in the MF-DS as studies investigating meats specifically suggest animal protein to be an important factor in stimulating muscle protein synthesis and preserving skeletal muscle mass (37, 38). Adherence to the two dietary indices (represented by scoring) is presented for the CALM study population in Table 3.

**Table 3:** Scoring according to dietary indices in the CALM study population.

**Dietary index**	**Median (IQR)**		
	**Men (*n* = 98)**	**Women (*n* = 86)**	**All (*n* = 184)**
BMD-DS	5 (5)	7 (4)	6 (5)
MF-DS	8 (4)	10 (4)	9 (4)

*BMD-DS, Bone Mineral Density-Diet Score; MF-DS, Muscle and Functional-Diet Score; IQR, inter quartile range (Q3–Q1)*.

#### Associations Between Adherence to Dietary Indices and BMD and Muscle Function in the CALM Study Population

The investigated associations between the BMD-DS and four different BMD outcomes in the CALM study population are presented in Table 4. No statistically significant associations were found between adherence to BMD-DS and the included BMD measures in any of our analyses. Adjusting for calcium intake did not influence any of the investigated associations (results not shown).

**Table 4:** Associations between BMD-Diet Score (BMD-DS) and BMD outcomes in the CALM study population.

	**Whole body BMD**						**Femur neck BMD**					
	**Model 1**		**Model 2**		**Model 3**		**Model 1**		**Model 2**		**Model 3**	
	**β**	**95% CI**	**β**	**95% CI**	**β**	**95% CI**	**β**	**95% CI**	**β**	**95% CI**	**β**	**95% CI**
Per SD	−0.005	(−0.022, 0.013)	−0.003	(−0.019, 0.013)	0.004	(−0.014, 0.023)	0.006	(−0.014, 0.027)	0.005	(−0.014, 0.024)	0.016	(−0.005, 0.038)
Q1 vs. Q2	−0.004	(−0.052, 0.042)	−0.009	(−0.052, 0.032)	−0.008	(−0.050, 0.034)	0.011	(−0.040, 0.061)	0.004	(−0.044, 0.051)	0.007	(−0.040, 0.055)
Q1 vs. Q3	0.001	(−0.050, 0.052)	0.009	(−0.037, 0.051)	0.013	(−0.034, 0.061)	0.023	(−0.034, 0.080)	0.024	(−0.029, 0.077)	0.033	(−0.022, 0.088)
Q1 vs. Q4	−0.032	(−0.092, 0.026)	−0.008	(0.062, 0.046)	0.002	(−0.057, 0.053)	0.004	(−0.063, 0.071)	0.022	(−0.041, 0.086)	0.034	(−0.031, 0.098)
*P for trend*	0.621		0.717		0.630		0.528		0.591		0.129	
	**Femur total BMD**						**L1–L4 BMD**					
	**Model 1**		**Model 2**		**Model 3**		**Model 1**		**Model 2**		**Model 3**	
	**β**	**95% CI**	**β**	**95% CI**	**β**	**95% CI**	**β**	**95% CI**	**β**	**95% CI**	**β**	**95% CI**
Per SD	0.006	(−0.015, 0.027)	0.007	(−0.013, 0.027)	0.017	(−0.006, 0.039)	−0.024	(−0.056, 0.008)	−0.027	(−0.058, 0.004)	−0.007	(−0.042, 0.027)
Q1 vs. Q2	0.016	(−0.037, 0.069)	0.011	(−0.038, 0.062)	0.014	(−0.036, 0.064)	0.038	(−0.044, 0.119)	0.026	(−0.053, 0.104)	0.032	(−0.046, 0.110)
Q1 vs. Q3	0.011	(−0.049, 0.070)	0.015	(−0.041, 0.072)	0.022	(−0.036, 0.080)	−0.067	(−0.158, 0.025)	−0.071	(−0.159, 0.017)	−0.055	(−0.145, 0.035)
Q1 vs. Q4	0.001	(−0.070, 0.070)	0.025	(−0.041, 0.092)	0.034	(−0.034, 0.102)	−0.062	(−0.167, 0.044)	−0.044	(−0.146, 0.059)	−0.021	(−0.125, 0.083)
*P for trend*	0.590		0.506		0.145		0.145		0.085		0.672	

*Coefficients and corresponding 95% CI for the linear modeling of the BMD Diet Score and bone mineral density in the CALM study population. Results are presented per SD and per quartile with Q1 as the reference. Model 1 is adjusted for age, sex and total energy intake. Model 2 is adjusted for model 1 + body weight and height. Model 3 is adjusted for model 2 + physical activity level and alcohol intake. Statistical significance is indicated by bold numbers, p < 0.05. Q, Quartiles; SD, Standard Deviation; CI, Confidence Interval; BMD, Bone Mineral Density*.

The investigated associations between the MF-DS and outcomes related to muscle function are presented in Table 5. We found an inverse association between adherence to the MF-DS and 400 m-gt both in the linear analyses (model 3 results: β = −5.4, 95 CI: −8.6, −0.7) and when comparing Q1 vs. Q3 (model 3 results: β = −11.3, 95 CI: −22.4, −0.3). Furthermore, we observed a positive association between adherence to the MF-DS and 30 s-cs when comparing Q1 vs. Q2 (β = 2.1, 95 CI: 0.4, 3.7).

**Table 5:** Associations between Muscle and Functional Diet Score (MF-DS) and outcomes related to muscle function in the CALM study population.

	**Grip strength**						**Knee extensor MVC**					
	**Model 1**		**Model 2**		**Model 3**		**Model 1**		**Model 2**		**Model 3**	
	**β**	**95% CI**	**β**	**95% CI**	**β**	**95% CI**	**β**	**95% CI**	**β**	**95% CI**	**β**	**95% CI**
Per SD	0.912	(−0.079, 1.903)	0.892	(−0.08, 1.864)	0.690	(−0.321, 1.701)	2.224	(−2.771, 7.219)	2.392	(−2.491, 7.274)	2.386	(−2.722, 7.494)
Q1 vs. Q2	0.231	(−2.317, 2.778)	0.021	(−2.474, 2.515)	−0.173	(−2.670, 2.324)	11.869	(−0.860, 24.598)	10.925	(−1.547, 23.396)	10.761	(−1.825, 23.347)
Q1 vs. Q3	1.098	(−1.702, 3.899)	1.239	(−1.509, 3.987)	0.661	(−2.175, 3.497)	−1.360	(−15.249, 12.529)	0.031	(−13.597, 13.658)	−0.564	(−14.753, 13.626)
Q1 vs. Q4	−0.705	(−3.950, 2.539)	−0.247	(−3.481, 2.987)	−0.796	(−4.080, 2.489)	−2.677	(−18.765, 13.410)	1.352	(−14.685, 17.388)	1.105	(−15.329, 17.540)
*P for trend*	0.071		0.072		0.180		0.381		0.335		0.358	
	**400 m gait time**						**30 s chair stands**					
	**Model 1**		**Model 2**		**Model 3**		**Model 1**		**Model 2**		**Model 3**	
	**β**	**95% CI**	**β**	**95% CI**	**β**	**95% CI**	**β**	**95% CI**	**β**	**95% CI**	**β**	**95% CI**
Per SD	−6.433	**(−10.740**, **−2.126)**	−5.421	**(−9.308**, **−1.535)**	−4.682	**(−8.642**, **−0.722)**	0.4	(−0.316, 1.115)	0.393	(−0.25, 1.035)	0.272	(−0.396, 0.941)
Q1 vs. Q2	−10.762	(−21.642, 0.118)	−10.112	**(−19.973**, **−0.252)**	−9.355	(−19.062, 0.353)	1.916	**(0.108, 3.723)**	2.170	**(0.559, 3.781)**	2.060	**(0.444, 3.676)**
Q1 vs. Q3	−17.244	**(−29.208**, **−5.280)**	−14.427	**(−25.289**, **−3.565)**	−11.34	**(−22.366**, **−0.313)**	0.427	(−1.560, 2.414)	0.120	(−1.655, 1.894)	−0.208	(−2.044, 1.627)
Q1 vs. Q4	−17.752	**(−31.610**, **−2.894)**	−10.253	(−23.035, 2.530)	−10.166	(−22.937, 2.604)	1.581	(−0.721, 3.883)	0.650	(−1.439, 2.738)	0.339	(−1.787, 2.464)
*P for trend*	**0.004**		**0.007**		**0.021**		0.272		0.229		0.423	

*Coefficients and corresponding 95% CI for the linear modeling of the Muscle Diet Score and muscle strength and functional outcomes in the CALM study population. Results are presented per SD and per quartile with Q1 as the reference. Model 1 is adjusted for age, sex and total energy intake. Model 2 is adjusted for model 1 + body weight and height. Model 3 is adjusted for model 2 + physical activity level and alcohol intake. Statistical significance is indicated by bold numbers, p < 0.05. Q, Quartiles; SD, Standard Deviation; CI, Confidence Interval; MVC, Maximum Voluntary Contraction*.

Adherence to MF-DS was positively associated with lean body mass only when comparing Q1 vs. Q3 (model 3 results: β = 1.6, 95 CI: 0.3, 3.0). This was also seen for adherence to the BMD-DS (model 3 results: Q1 vs. Q2, β = 1.7, 95 CI: 0.3, 3.0 and Q1 vs. Q3, β = 1.3, 95 CI: 0.5, 2.9).

As the only difference between the BMD-DS and the MF-DS was the two food groups “red and processed meats” and “confectionery” (included as negative contributors in the BMD-DS, but not in the MF-DS), BMD-DS and MF-DS were interchanged and all linear analyses were performed again. As was the case for BMD-DS, we found no associations between MF-DS and any of the BMD outcomes in model 3 and 400 m-gt was again the only consistent outcome inversely associated with increased adherence (results not shown). No interactions were seen between dependent and independent variables for either sex, age, or physical activity.

Adjusting for total protein intake in our analyses attenuated the associations only very slightly (exemplified by the per SD association between MF-DS adherence and 400 m-gt changing only from β = −4.7, *p* = 0.021 to β = −4.6, *p* = 0.027, model 3). Thereby indicating that increased protein intake was not a major explanatory factor in the observed association between MD-DS and 400 m-gt. Adjusting for the dietary groups individually demonstrated that controlling for fruit intake attenuated the association and statistical significance between MF-DS adherence and 400 m-gt (β = −3.2, *p* = 0.18, model 3). No significant changes were seen when adjusting for other food groups.

## Discussion

In the present study, we updated the narrative review and the BMD-Diet Score created by de Jonge et al. (13). The additional studies published since this review resulted in only minor changes to the BMD-Diet Score.

We used the same narrative review approach to produce a similar dietary scoring index (MF-DS) containing the food groups that were most often associated with muscle function in previously published studies. As evident from Figures 1, 2, the two dietary indices were similar. The only difference was that the “red and processed meats” (purposely excluded from the MF-DS) and the “confectionery” food groups were included as negative contributors in the BMD-DS, but not in the MF-DS. No differences were seen when interchanging the two indices in any our analyses. Neither adherence to BMD-DS nor MF-DS showed associations with BMD in our study population and adherence to both indices were inversely associated with 400 m-gt and positively associated with 30 s-cs (only in the Q1 vs. Q2 comparisons). No associations were observed with other muscle outcomes. Hence, contrary to expectations, neither of the indices could explain BMD variation in our study population of relatively healthy and active older Danes. Yet, they were equally good at explaining outcomes related to muscle function (mobility).

Since an association between the BMD-DS and BMD was previously found in a Dutch cohort of older adults (13), the current findings may potentially be explained by generally high BMD levels in our study population. It is possible that the intake of these “bone healthy” foods is more effective in people with lower BMD or that lack of adequate statistical power prevented us from replicating previous findings. The latter consideration may very well be true since the associations observed in the current study are similar to the ones from the de Jonge et al. study (despite the statistical differences). As an example, we observed a per SD β-value of 0.005 (95% CI:−0.014; 0.027) for the association between BMD-DS adherence and femoral neck BMD. For a similarly adjusted model, this association was β = 0.009 (95% CI: 0.005; 0.012) in the de Jonge et al. study. Alternatively, these foods are not as “bone healthy” as suggested previously. Future studies should investigate associations (or effects) of adherence to similar indices in larger sample sizes preferably with a large variation in baseline BMD measures to establish if baseline BMD status is influencing the “effectiveness” of these foods.

Our results indicate that adhering to dietary indices based on higher intakes of whole grains, dairy products, fish, legumes, nuts, fruit, and vegetables are associated with faster 400 m walking speeds and an increased number of chair stands measured over a 30-s time period. Adherence to a dietary pattern closely resembling that of many dietary recommendations could result in sustained muscle strength and function, partly due to ample amounts of micronutrients, bioactive compounds and quality protein that previously have been associated with reduced rates of age-related muscle loss (11) as well as aging of the brain and nervous system (39, 40), factors that potentially could affect functional capability, particularly in older individuals. To examine the influence of dietary protein on the observed associations between adherence to our indices and functional outcomes, we adjusted for total protein intake in our analyses. This adjustment attenuated the associations only slightly, indicating that increased protein intake was not a major determinant of the investigated outcomes. Adjusting for the dietary groups individually demonstrated that fruit intake attenuated the association and statistical significance for 400 m gait time. This could indicate that this food group could be of relevance in relation to sustaining functional capabilities of older individuals.

As BMD-DS and MF-DS were similarly associated with our outcomes, the two food groups distinguishing the two indices (“red and processed meats” and “confectionery”) were evaluated as less important for these outcomes. Previous studies have suggested intake of animal protein to be beneficial in regards to skeletal muscle mass (37, 41) and potentially also prevention of osteoporosis (42, 43). Unfortunately, intake of red and processed meats are strongly associated with increased risk of adverse health outcomes such as colorectal cancers (44). However, we suspect that the intake of red and processed meats is not necessary to sustain muscle mass and function in old age as long as adequate protein intake is reached via other protein rich food sources such as e.g., poultry, dairy products, cereals, legumes, and nuts.

In the current study, adhering to a dietary index containing whole grain, dairy products, fish, legumes, nuts, fruit, and vegetables were not established as important in relation to BMD in our study group of community-dwelling older adults. In contrast, such associations were found in a similarly aged Dutch population by de Jonge et al. The influence of these food groups in relation to BMD needs to be further investigated.

The development of a MDS with established food groups beneficial for both BMD and muscle function was not possible in the current study. Nonetheless, considering the results of our narrative review, it is likely that a large overlap exists between foods that are potentially beneficial for BMD and muscle function. Future studies should investigate whether adherence to a dietary index including these food groups is associated with BMD and muscle function in other populations, or alternatively produce novel dietary indices using data-driven approaches such as reduced rank regression or similar.

The current study is strengthened by the inclusion of several measures related to BMD, muscle strength and functional capability in older individuals. The inclusion of these measures enabled a thorough investigation into potential associations between diet, BMD, and muscle function. A limitation of the current study is its cross-sectional design that entails an inherent risk of reverse causality. Also, we assessed food intake using 3 days weighed dietary records, which may not be sufficient to reach a robust insight into the habitual food intake of our study population. However, study participants not consuming, for instance, dairy or whole grain products within the registration period do likely not consume such foods on a regular basis and would therefore correctly be placed in the “low-intake group” independent of the dietary registration method. A minor change was introduced to our dietary index, compared to the de Jonge review, as intake of beans was too low for any sensible analysis. The food group “legumes and beans” was changed to “legumes and nuts” in order to reach intake levels that enabled meaningful analyses. Nuts and beans share similar properties in relation to, for instance, their content of complex carbohydrates, dietary fibers, vitamins, and minerals. It was therefore the most reasonable substitution feasible. As the CALM study is a randomized intervention trial, information on e.g., social-status, smoking habits, and other potential confounders were not registered. Confounding from these sources can therefore not be excluded. Our study includes a relatively small sample size of 184 well-functioning Danish elderly. This is relevant to consider in terms of our study's statistical power as well as the generalizability of our results. Lastly, it is important to recognize that a narrative review approach is limited by which dietary patterns have previously been investigated in relation to BMD and muscle outcomes. Thus, the current approach does not necessarily answer the question “what is the optimal diet in relation to BMD and muscle function in older adults?” The current approach does nonetheless give an insight into which food groups that are most often associated with BMD and muscle function and therefore could be part of a diet supporting a healthy aging process.

## Conclusion

In the current study we updated a previously conducted narrative review on food groups associated with BMD and the resulting BMD-Diet Score. Our update did not lead to changes in the originally proposed BMD-Diet Score. Secondly, we conducted a narrative review of food groups associated with muscle strength and functional outcomes and constructed a so-called Muscle and Functional-Diet Score. The food groups included were highly similar for the two dietary scores. Lastly, we investigated associations between adherence to the two dietary scores and outcomes related to BMD and muscle strength, mass, and function in a group of older Danes. Our results showed that adhering to dietary scores based on high intakes of whole grains, dairy products, legumes, nuts, fish, fruit, and vegetables is associated with faster walking speeds and an increased number of chair stands measured over a 30 s time period. We were unable to reproduce earlier findings, which demonstrated that adherence to the BMD-Diet Score was associated with changes in BMD. Future studies should investigate whether adherence to such dietary patterns are associated with BMD and muscle outcomes in other populations of older individuals.

## Data Availability

The datasets used and analyzed during the present study are available from the corresponding author on request.

## Ethics Statement

The study was approved by the Danish Regional Ethical Committees of the Capital Region (J-nr. H-4-2013-070) and all participants gave written informed consent in accordance with the Declaration of Helsinki.

## Author Contributions

SS, ML, and IT conceived and designed the study, drafted the manuscript, and read and approved the final version. KM, JB, RB, GH, AS, MH, CS, SA, MJ, SR, and LH drafted the manuscript and read and approved the final version.

### Conflict of Interest Statement

The authors declare that the research was conducted in the absence of any commercial or financial relationships that could be construed as a potential conflict of interest.
