# Supplementary material for: Development of a Mobility Diet Score (MDS) and Associations With Bone Mineral Density and Muscle Function in Older Adults
Source: Front Nutr. 2019 Sep 4;6:114. doi: 10.3389/fnut.2019.00114 (PMC6738326; doi:10.3389/fnut.2019.00114)
Supplement: Supplementary file 1 [file Table_1.DOCX]

| **S1.** Studies investigating dietary patterns positively associated with BMD | | | |  |
| --- | --- | --- | --- | --- |
| **Reference** | **Sample Size** | **Design,  Dietary Data Assessment** | **Food Groups and their Factor Loadings** | |
|  |  |  | **Food Groups** | **Factor  Loadings** |
| Rogers, Osteoporosis int., 2018 (26) | 4218 (100% men) | Cohort study,  Block 98.2 FFQ | **"Prudent" dietary pattern** |  |
|  |  |  | Baked beans, pintos | 0.43 |
|  |  |  | Broccoli | 0.56 |
|  |  |  | Carrots | 0.61 |
|  |  |  | Coleslaw, cabbage | 0.44 |
|  |  |  | Corn | 0.38 |
|  |  |  | Dark bread | 0.31 |
|  |  |  | Green beans | 0.49 |
|  |  |  | Fish (not fried) | 0.43 |
|  |  |  | Other vegetables | 0.47 |
|  |  |  | Rice | 0.32 |
|  |  |  | Green salad | 0.47 |
|  |  |  | Spinach | 0.50 |
|  |  |  | Sweet potatoes | 0.32 |
|  |  |  | Tofu | 0.36 |
|  |  |  | Raw tomatoes | 0.43 |
|  |  |  | Vegetable soup | 0.38 |
|  |  |  | Yogurt, frozen yogurt | 0.30 |
| Melaku,  Br. J. Nutr.,  2016 (22) | 1182 (45.9% men) | Retrospective cohort study, FFQ | **"Prudent" dietary pattern** |  |
|  |  |  | Legumes | >0.30 |
|  |  |  | Fish | >0.30 |
|  |  |  | Tea and water | >0.30 |
|  |  |  | Nut-based milk | >0.30 |
|  |  |  | Medium fat dairy | >0.30 |
|  |  |  | Sugar | >0.30 |
|  |  |  | Cabbages | >0.30 |
|  |  |  | Root vegetables | >0.30 |
|  |  |  | Other fruits | >0.30 |
|  |  |  | Stalk vegetables | >0.30 |
|  |  |  | Leafy vegetables | >0.30 |
|  |  |  | Fruity vegetables | >0.30 |
|  |  |  | White bread | >0.30 |
| Denova-Guitiérrez, Osteoporosis int., 2016 (23) | 6915  (28.2% men) | Cross-sectional study, FFQ | **"Prudent" dietary pattern** |  |
|  |  |  | Refined grains | -0.55 |
|  |  |  | Fresh vegetables | 0.68 |
|  |  |  | Tomato | 0.52 |
|  |  |  | Potatoes | 0.36 |
|  |  |  | Fresh fruits | 0.53 |
|  |  |  | Legumes | 0.28 |
|  |  |  | Oils | 0.40 |
|  |  |  | **"Dairy and fish" dietary pattern** |  |
|  |  |  | Corn tortilla and mexican food | -0.65 |
|  |  |  | Whole grains | 0.38 |
|  |  |  | Soft drinks | -0.32 |
|  |  |  | Fish and other seafood | 0.45 |
|  |  |  | Milk | 0.30 |
|  |  |  | Dairy foods | 0.45 |
|  |  |  | Legumes | -0.46 |
| Ward, J. Bone. Miner. Res.,  2016 (24) | 1263 (47.7% men) | Cohort study,  Food diaries | **"Protein-Calcium-Potassium-rich" dietary pattern** |  |
|  |  |  | Low fat milk | >0.30 |
|  |  |  | Animal-based fats | >-0.30 |
|  |  |  | White bread | >-0.30 |
|  |  |  | Sugar and preserves | >-0.30 |
| Park et al., Osong Public Health Res Perspect., 2012 (25) | 1725  (0% men) | Cohort study, FFQ | **"Dairy" dietary pattern** |  |
|  |  |  | Milk | 0.55 |
|  |  |  | Green tea and other drinks | 0.53 |
|  |  |  | Dairy products | 0.50 |
|  |  |  | Mushrooms | 0.43 |
|  |  |  | Seaweeds | 0.34 |
|  |  |  | Fish and seafoods | 0.32 |
|  |  |  | Rice and rice cake | -0.47 |
|  |  |  | Kimchi | -0.30 |

| **S2**. Studies investigating dietary patterns negatively associated with BMD | | | |  |
| --- | --- | --- | --- | --- |
| **Reference** | **Sample Size** | **Design,  Dietary Data Assessment** | **Food Groups and their Factor Loadings** | |
|  |  |  | **Food Groups** | **Factor  Loadings** |
| Denova-Guitiérrez, Osteoporosis int., 2016 (23) | 6915 (28.2% men) | Cross-sectional study, FFQ | **"Refined foods" dietary pattern** |  |
|  |  |  | Refined grains | 0.30 |
|  |  |  | Sugar and sweets | 0.58 |
|  |  |  | Soft drinks | 0.35 |
|  |  |  | Other beverages | -0.35 |
|  |  |  | Eggs | 0.35 |
|  |  |  | Red meat | 0.69 |
|  |  |  | Milk | -0.41 |
|  |  |  | Fats | 0.58 |
| Melaku,  Br. J. Nutr.,  2016 (22) | 1182 (45.9% men) | Cohort study, FFQ | **"Western" dietary pattern** |  |
|  |  |  | White bread | >0.30 |
|  |  |  | Soft drinks | >0.30 |
|  |  |  | Beer | >0.30 |
|  |  |  | Tea and water | >0.30 |
|  |  |  | Unsaturated spread | >0.30 |
|  |  |  | Red meat | >0.30 |
|  |  |  | Jam and vegemite | >0.30 |
|  |  |  | Take away foods | >0.30 |
|  |  |  | Snacks | >0.30 |
|  |  |  | Sugar | >0.30 |
|  |  |  | Processed meat | >0.30 |
| De Franca, Eur. J.  Clin. Nutr., 2016 (20) | 156 (0% men) | Cross-sectional study 3-day food diaries | **"Sweet foods, coffee and tea"  dietary pattern** |  |
|  |  |  | Sweet foods | 0.74 |
|  |  |  | Coffee and tea | 0.82 |
| Park et al., Osong Public Health Res Perspect., 2012 (25) | 1725  (0% men) | Cohort study, FFQ | **"Traditional" dietary pattern** |  |
|  |  |  | Potatoes | 0.42 |
|  |  |  | Legumes and nuts | 0.47 |
|  |  |  | Kimchi | 0.52 |
|  |  |  | Vegetables | 0.71 |
|  |  |  | Mushrooms | 0.32 |
|  |  |  | Fruits | 0.51 |
|  |  |  | Meat | 0.43 |
|  |  |  | Eggs | 0.40 |
|  |  |  | Fish and seafood | 0.56 |
|  |  |  | Seaweeds | 0.55 |
|  |  |  | **"Western" dietary pattern** |  |
|  |  |  | Noodles | 0.56 |
|  |  |  | Bread | 0.61 |
|  |  |  | Sugar and fat | 0.60 |
|  |  |  | Coffee and carbonated beverages |  |
|  |  |  | Meat | 0.35 |
|  |  |  | Fish and seafood | 0.30 |
| Wu,  Br. J. Nutr.,  2017 (21) | 347 (0% men) | Cross-sectional study,  FFQ | **"Processed Foods"  dietary pattern** |  |
|  |  |  | Dark yellow vegetables | 0.42 |
|  |  |  | Cruciferous vegetables | 0.42 |
|  |  |  | Red meats | 0.76 |
|  |  |  | Poultry | 0.71 |
|  |  |  | Processed meats | 0.51 |
|  |  |  | Fish | 0.32 |
|  |  |  | Potatoes | 0.50 |
|  |  |  | Chips | 0.31 |

| **S3.** Studies investigating dietary patterns positively associated with muscle function | | | |  |
| --- | --- | --- | --- | --- |
| *Exploratively derived dietary patterns* | | | | |
| **Reference** | **Sample Size** | **Design,  Dietary Data Assessment** | **Food Groups & Factor Loadings** |  |
|  |  |  | **Food Groups** | **Factor  Loadings** |
| Mohseni,  Aging Clin. Exp. Res., 2017 (27) | 250 (0% men) | Cross-sectional study,  FFQ | **"Mediterranean diet"  dietary pattern** |  |
|  |  |  | Legumes | 0.35 |
|  |  |  | Olive, olive oil | 0.65 |
|  |  |  | Low fat dairy | 0.61 |
|  |  |  | Vegetable | 0.60 |
|  |  |  | Fish | 0.59 |
|  |  |  | Nuts | 0.51 |
|  |  |  | Vegetable oil | 0.40 |
|  |  |  | Fruit | 0.40 |
|  |  |  | Egg | 0.34 |
| Chan, J. Am. Med. Dir. Assoc., 2016 (28) | 3667 (48.9% men) | Cohort study, FFQ | **"Vegetables-fruits" dietary pattern** |  |
|  |  |  | Other vegetables | 0.58 |
|  |  |  | Tomatoes | 0.49 |
|  |  |  | Dark green and leafy  vegetables | 0.43 |
|  |  |  | Cruciferous vegetables | 0.43 |
|  |  |  | Starchy vegetables | 0.42 |
|  |  |  | Soy | 0.42 |
|  |  |  | Fruits | 0.4 |
|  |  |  | Legumes | 0.34 |
|  |  |  | Fats and oils | -0.37 |
|  |  |  | **"Snacks-drinks-milk  products" dietary pattern** |  |
|  |  |  | Condiments | 0.48 |
|  |  |  | Coffee | 0.42 |
|  |  |  | Fast food | 0.37 |
|  |  |  | Nuts | 0.37 |
|  |  |  | French fries and potato  chips | 0.37 |
|  |  |  | Milk and milk products | 0.31 |
|  |  |  | Whole grains | 0.3 |
|  |  |  | Beverages | 0.22 |
|  |  |  | Refined grains | -0.5 |
| Hashemi, Nutr.,  2015 (29) | 300  (100% men) | Cross-sectional study, FFQ | **"Mediterranean diet" dietary pattern** |  |
|  |  |  | Olive | 0.72 |
|  |  |  | Low carotenoid vegetables | 0.6 |
|  |  |  | Whole grains | 0.59 |
|  |  |  | Dried fruits | 0.59 |
|  |  |  | Tomatoes | 0.57 |
|  |  |  | Nuts | 0.54 |
|  |  |  | High carotenoid vegetables | 0.54 |
|  |  |  | High carotenoid fruit | 0.54 |
|  |  |  | Pickles | 0.46 |
|  |  |  | Fish | 0.44 |
|  |  |  | Low carotenoid fruit | 0.44 |
| *Pre-defined dietary patterns* | | | | |
| **Reference** | **Sample Size** | **Design,  Dietary Data Assessment** | **Definition of dietary pattern** | |
| Tian,  Br. J. Nutr.,  2017 (30) | 3289 (32.2% men) | Cross-sectional study,  FFQ | **Alternate Mediterranean Diet** High in whole grain, vegetables, fruits, legumes, nuts, fish and MUFA:SFA, low in meat and moderate alcohol intake | |
| Perälä,  Age Ageing,  2017 (13) | 1072 (44% men) | Cohort study, FFQ | **The Healthy Nordic Diet** High in Nordic fruits, vegetables, cereals, fish low-fat milk, PUFA:SFA, low in red & processed meats and moderate alcohol intake | |
| Mangano,  Am. J. Clin. Nutr.,  2017 (31) | 2986 (46% men) | Cohort study,  FFQ | **Dietary Protein Patterns** High in legumes, low-fat milk, chicken, red meat, fish, fast-food, full-fat diary | |
| Kelaiditi,  Osteoporosis int.,  2016 (12) | 2570 + 949 (0% men) | Cross-sectional study, FFQ | **Mediterranean Diet**  High in vegetables, legumes, fruits, nuts, cereal, fish & MUFA:SFA, low in meat, poultry & high-fat diary products and moderate alcohol intake | |
| León-Muñoz,  J. Am. Med. Dir.  Assoc., 2014 (32) | 1815 (43% men) | Cohort study, Validated computerized diet history | **Mediterranean Diet** MDS: High in whole grain, vegetables, fruits, legumes, nuts, fish, MUFA:SFA, low in red & processed meats, moderate alcohol intake  MEDAS: High in olive oil, fruit, vegetables, legumes, nuts, fish, shellfish, sofrito, low intake of red meats, butter/margarine/cream, sweet or carbonated drinks, sweets and cake, moderate wine intake | |
| Bollwein,  J. Gerontol A  Biol. Sci. Med.  Sci., 2013 (33) | 192 (35.4% men) | Cross-sectional study, FFQ | **Mediterranean Diet** High in whole grain, vegetables, fruits, legumes, nuts, fish, MUFA:SFA, low in red & processed meats, moderate alcohol intake | |
